# Hypomethylation of *MIR‐378* 5’‐flanking region predicts poor survival in young patients with myelodysplastic syndrome

**DOI:** 10.1002/mgg3.1067

**Published:** 2019-12-13

**Authors:** De‐hong Wu, Xiao‐wen Zhu, Xiang‐mei Wen, Ying‐ying Zhang, Ji‐chun Ma, Dong‐ming Yao, Jing‐dong Zhou, Hong Guo, Peng‐fei Wu, Xing‐li Zhang, Hong‐chun Qiu, Jiang Lin, Jun Qian

**Affiliations:** ^1^ Department of Hematology Affiliated People’s Hospital of Jiangsu University Zhenjiang Jiangsu People’s Republic of China; ^2^ Department of Hematology Kunshan Third People’s Hospital KunShan Jiangsu People’s Republic of China; ^3^ Laboratory Center Affiliated People’s Hospital of Jiangsu University Zhenjiang Jiangsu People’s Republic of China; ^4^ The Key Lab of Precision Diagnosis and Treatment in Hematologic Malignancies of Zhenjiang City Zhenjiang Jiangsu People’s Republic of China

**Keywords:** hypomethylation, *MIR‐378*, myelodysplastic syndrome, prognosis

## Abstract

**Background:**

Previous studies have disclosed up‐regulation of *MIR‐378* in acute myeloid leukemia (AML), and might consequently affect the outcome of the patients. Correspondingly, hypomethylation of *MIR‐378* was also identified in AML, particularly for FAB‐M2 subtype with t(8;21) chromosomal translocation. Nevertheless, the methylation status of *MIR‐378* has not been illustrated in myelodysplastic syndrome (MDS). Herein we designed to understand the methylation pattern of *MIR‐378* involved in MDS and clinical interrelation thereof.

**Methods:**

Real‐time quantitative methylation‐specific PCR (RQ‐MSP) was performed to evaluate the methylation degree of *MIR‐378* 5’‐flanking region on bone marrow mononuclear cells collected from 95 de novo MDS patients. Five gene mutations (*IDH1*, *IDH2*, *DNMT3A*, *U2AF1*, and *SF3B1*) were detected by high‐resolution melting analysis to further evaluate the clinical relevance of hypomethylation of *MIR‐378*.

**Results:**

Unmethylated level of *MIR‐378* 5’‐flanking region was significantly higher in MDS patients than that in controls (*p* = .034). Hypomethylated *MIR‐378* was identified in 20 of 95 (21%) cases with MDS. Male patients appeared to be more frequent to harbor *MIR‐378* hypomethylation compared to female patients (15/55, 27.3% vs. 4/40, 10.0%, *p = *.04). There was no significant difference in age, white blood cell counts, platelet counts, hemoglobin concentration, and karyotypes between the patients with and without *MIR‐378*‐hypomethylation. Distinct distribution of five gene mutations was not observed in the two groups as well. However, *MIR‐378*‐hypomethylated patients had significantly shorter overall survival than those without *MIR‐378* hypomethylation (*p = *.036). Moreover, among patients <60 years, hypomethylation of *MIR‐378* was confirmed to be an independent adverse prognostic factor by both Kaplan–Meier and Multivariate Cox analyses.

**Conclusion:**

Hypomethylation of *MIR‐378* 5’‐flanking region is an adverse prognosticator in MDS, particularly in patients <60 years.

## Graphical abstract

Hypomethylation level of *MIR‐378* was significantly higher in myelodysplastic syndrome (MDS) patients than that in controls (*p* = .034). *MIR‐378*‐hypomethylated patients had significantly shorter overall survival than those without *MIR‐378* hypomethylation (*p* = .036). Both Kaplan–Meier and Multivariate Cox analyses confirmed that hypomethylation of *MIR‐378* 5’‐flanking region is an adverse prognosticator in MDS, particularly in patients <60 years.

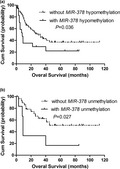

## 1 INTRODUCTION

Myelodysplastic syndrome (MDS) is a hematopoietic malignance characterized by dyshematopoiesis with a high tendency to transformation of acute myeloid leukemia (AML) (Valent et al., 2007). Cytogenetic as well as genetic alterations have been widely illustrated and considered to play a vital role in the processes of MDS (Adès, Itzykson, & Fenaux, 2014; Lee, Podoltsev, Gore, & Zeidan, 2015). Meanwhile, epigenetic variations, particularly for the aberrant DNA methylation, were also deemed to participate in the development of MDS (Issa, 2013; Itzykson & Fenaux, 2014). In spite of promising clinical application of hypomethylating agents (HMAs) presented recently, however, primary drug resistance and recurrence or disease progress during the treatment are the main obstacles (Carella, 2015), the outcome of patients with MDS generally remains dismal. The knowledge about pathogenesis of MDS is still insufficient. Until recently, a group of small noncoding RNA molecules (19–24 nts), microRNAs, have emerged as crucial roles in nearly all kinds of biologic functions such as development, differentiation, apoptosis, and proliferation (Schmittgen, 2008; Winter, Jung, Keller, Gregory, & Diederichs, 2009). Ectopic expression of miRNAs was implicated in a wide scale of diseases including cancers (Barbarotto, Schmittgen, & Calin, 2008; Calin & Croce, 2006). Increasing number of studies have even supposed that deregulation of various microRNAs contributed to pathogenesis of MDS and transformation into AML (Pons et al., 2009; Votavova et al., 2011), furthermore, large number of microRNAs could be served as useful biomarkers to predict the outcome (Bhise et al., 2016; Cattaneo et al., 2015; Xu, Guo, Zhang, Chen, & Hu, 2016). Strikingly, researchers have reported distinctive influence of *MIR‐378* in many cancers, such as lung cancer (Chen et al., 2012), colorectal carcinoma (Faltejskova et al., 2012), gastric cancer (Deng et al., 2013). Our previous study preliminarily observed overexpressed *MIR‐378* was often related to AML and exerted adverse effects on prognosis (Qian et al., 2013). Correspondingly, hypomethylated *MIR‐378* was revealed to be associated with AML, especially in FAB‐M2 subtype (Xiao‐Wen et al., 2015). However, there is limited information about *MIR‐378* methylation status in MDS. This work was to determine the methylation status of the 5’‐flanking region of *MIR‐378,* which contains 212 CpG dinucleotides, and further to analyse the clinical role in MDS.

## 2 MATERIALS AND METHODS

### 2.1 Clinical samples

This work was approved by both the Ethics Committees of the Affiliated People's Hospital of Jiangsu University and Kunshan Third People's Hospital. A total of 95 primary MDS patients, treated at the Affiliated People’ Hospital of Jiangsu University or Kunshan Third People's Hospital were included in this study. All bone marrow (BM) samples were obtained at initial diagnosis. Written informed consents were signed by each participant before the BM samples were collected. BM samples from 16 health donors were served as controls. The diagnosis of each case was established according to World Health Organization (WHO) and French‐American‐British (FAB) classification (Bennett et al., 2010; Vardiman et al., 2009). The risk classification for each patient was on the basis of International Prognosis Scoring System (IPSS) for MDS (Greenberg et al., 1997). The clinical features as well as laboratory tests of whole MDS patients were displayed in Table 1.

**Table 1:** Correlation of *MIR‐378* hypomethylation with clinical features in MDS

Clinical parameters	*MIR‐378* hypomethylation			
	No (*n* = 75)	Yes (*n* = 20)	Total (*n* = 95)	*p*
Age (years)^a^	60 (20–86)	63.5 (27–85)	60 (20–86)	.560
Sex (male/female)	39/36	16/4	55/40	.040
WBC (×10^9^/L)^a^	2.8 (0.9–26.6)	2.4 (1.3–19.5)	2.7 (0.9–26.6)	.204
HB (g/L)^a^	68 (26–128)	58 (29–104)	63.5 (26–118)	.088
PLT (×10^9^/L)^a^	63 (0–754)	60 (9–1176)	61.5 (0–1176)	.964
Cytogenetics				.607
Good	54	15	69	
Intermediate	9	1	10	
Poor	5	3	8	
No data	7	1	8	
WHO classifications				.486
RARS	8	4	12	
RCMD (RS)	32	5	37	
5q‐	2	0	2	
RAEB‐1	16	4	20	
RAEB‐2	16	7	23	
MDS‐U	1	0	1	
FAB classifications				.222
RA	32	7	39	
RARS	15	2	17	
RAEB	26	11	37	
RAEBt	2	0	2	
IPSS				.541
Low	5	3	8	
Int‐1	45	9	54	
Int‐2	13	4	17	
High	8	2	10	
No data	4	2	6	
Gene mutations				
U2AF1 (+/−)	5/66	2/15	7/81	.616
IDH1/2 (+/−)	4/68	0/17	4/85	1.000
DNMT3A (+/−)	2/70	1/16	3/86	.475
SF3B1 (+/−)	5/66	1/16	6/82	1.000

Abbreviations: FAB, French‐American‐British; HB, hemoglobin; IPSS, International Prognostic Scoring System; MDS, myelodysplastic syndrome; PLT, platelet count; RA, refractory anemia; RAEB, RA with excess of blasts; RARS, RA with ringed sideroblasts; RCMD, refractory cytopenia with multilineage dysplasia; RCMD‐RS, RCMD with ringed sideroblasts; WBC, white blood cells; WHO, World Health Organization.

a Median (range).

### 2.2 Therapeutic regimen

Treatment options including supportive care, low‐ or high‐intensity therapies, were given generally according to the risk classification and the wishes of patients. Briefly, symptomatic Low/Int‐1 patients were managed with hematopoietic growth factors, thalidomide or immunosuppressive therapy, and supportive treatment. The treatment for Int‐2/High patients included decitabine or chemotherapy with CAG (aclacinomycin, cytarabine, and G‐CSF) or HAG (homoharringtonine, cytarabine, and G‐CSF) protocol.

### 2.3 DNA isolation, bisulfite modification

The BM mononuclear cells (BMNCs) were obtained by using of Ficoll‐Hypaque gradient. Genomic DNA extraction from the BMNCs was conducted via genomic DNA purification kit (Gentra), and then CpGenome^™^ DNA Modification Kit (Chemicon) was used to treat the production of genomic DNA according to the manufacturer's protocol.

### 2.4 Methylation‐specific PCR

The methylation‐specific PCR (MSP) reactions for methylated *MIR‐378* and unmethylated *MIR‐378* were performed on Step One Plus (Applied Biosystems) using two sets of methylated (M) and unmethylated (U) specific primers (Table 2). For the control, we chose *ALU* repetitive sequence as was described before (Qian et al., 2011). The amplification reaction system consisted of 10 μM SYBR Premix Ex Taq II, 0.4 μM of primers, 0.4 μL 50× ROX (Takara), and 20 ng of bisulfite‐modified DNA. Amplification for methylated specific primers was conducted in the conditions of 95°C for 30 s, cycled at 95°C for 5 s, 62°C for 30 s, and 72°C for 30, and a fluorescence collection step at 75°C for 30 s (40 cycles), followed by a melting program at 95°C for 15 s, 60°C for 60 s, 95°C for 15 s, and 60°C for 15 s. In real‐time quantitative MSP (RQ‐MSP) for unmethylated specific primers of *MIR‐378*, the amplification was carried out in the following program: 95°C for 30 s, then 40 cycles of 95°C for 5 s, 62°C for 30 s, 72°C for 30 s, and 75°C for 30 s (fluorescence collection step), followed by a melting program at 95°C for 15 s, 60°C for 60 s, 95°C for 15 s, and 60°C for 15 s.

**Table 2:** The sequences of primers used in RQ‐MSP and BSP

	Forward (5’→3’)	Reverse (5’→3’)	Product (bp)
RQ‐MSP			
M	GGATGAGTTTTGAGTCGTTT	CCATACAAACCGCTCACTCC	129
U	TAGGATGAGTTTTGAGTTGT	ATAATCCCATACAAACCACT	137
BSP	AGGATTTTTTGGTGATTTTTG	TCACCCTCTAACTACATAATCCC	200

Abbreviations: BSP, bisulfite‐sequencing PCR; RQ‐MSP, real‐time quantitative methylation‐specific PCR.

The normalized ratio (N_unmethylation‐_ *_MIR‐378_*) was used to evaluate unmethylated level of *MIR‐378*. N_unmethylation‐_ *_MIR‐378_* was determined using the 2^−ΔΔCT^ method comparing the *ALU* gene level. This means that a higher value of N_unmethylation‐_ *_MIR‐378_* represents a lower methylation status or hypomethylation of *MIR‐378*. The products of MSP were resolved in the concentration of 2% agarose gels to visualize under UV illumination.

### 2.5 Bisulfite‐sequencing PCR

In our experiment, Bisulfite‐sequencing PCR (BSP) was used to further determine DNA methylation density of *MIR‐378* in samples from three *MIR‐378*‐hypermethylated and three *MIR‐378* hypomethylated MDS patients according to the results of RQ‐MSP. The PCR was conducted with the reaction system containing 6.25 μM of dNTP mixture, 10× PCR buffer (0.25 mM KCl), 0.75 U of Hot start DNA polymerase (Takara), 0.5 μM of primers, and 20 ng of modified DNA on iCycler Thermal Cycler (Eppendorf). The bisulfite‐treated DNA was amplified with sequencing primers in Table 2. The conditions of amplification were 98°C for 10 s, then cycled at 98°C for 10 s, 56°C for 30 s, 72°C for 30 s, and entered to extension at 72°C for 7 min. After the program of purification, the PCR products were cloned into pMD^®^19‐T Vector (Takara), and 10 clones of each BSP product were sequenced (http://www.bgitechsolutions.cn/bbs). The percentage of methylation was calculated from the number of methylated CpG divided by total CpG loci of all 10 clones.

### 2.6 Mutations analysis

High‐resolution melting analysis (HRMA) (Lin et al., 2011, 2012; Qian et al., 2012; Yang et al., 2013) was conducted to analyze *IDH1, IDH2, DNMT3A, SF3B1, and U2AF1* gene mutations. Direct DNA sequencing was employed to verify the positive results.

### 2.7 Statistical analysis

The SPSS software 22.0 package was used for Statistical analysis. Mann–Whitney/Wilcoxon test was performed to compare two groups with continuous variables. For the comparison of categorical variables between patient teams, the statistical method utilized was Pearson Chi‐square analysis or Fisher exact test. Kaplan–Meier method was employed for survival analysis, and the identification of the independent risk factors affecting the survival was carried out by Cox regression model. A two‐tailed *p* < .05 was considered statistically significant.

## 3 RESULTS

### 3.1 Hypomethylation of *MIR‐378* 5’‐flanking region in MDS patients

**Figure 1:**
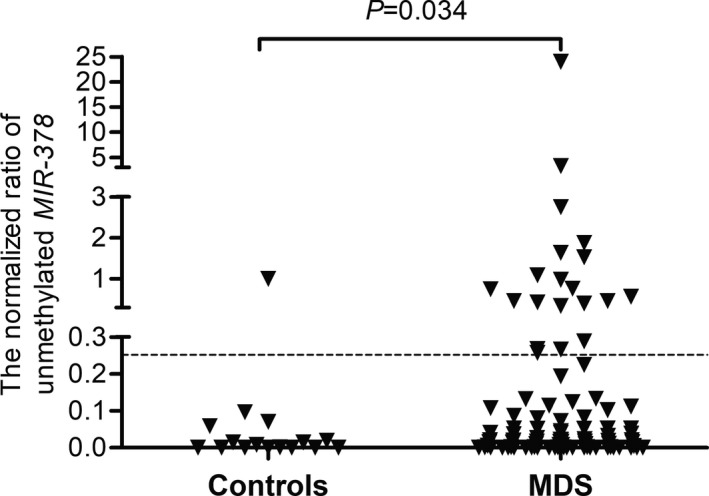
Relative Levels of *MIR‐378* unmethylation in MDS and control. The cutoff value of the mean plus 1 *SD* (0.252) in normal controls was displayed with the dotted line. MDS, myelodysplastic syndrome

**Figure 2:**
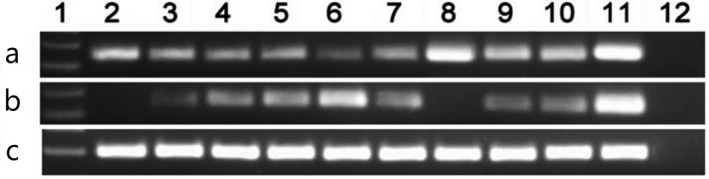
Electrophoresis results of RQ‐MSP products in MDS patients. (a) *MIR‐378* methylation; (b) *MIR‐378* unmethylation; (c) *ALU*. 1: Gene Ruler^™^ 100bp DNA ladder; 2–3: normal controls; 4–10: MDS samples; 11: cloned plasmid; 12: negative control. MDS, myelodysplastic syndrome; RQ‐MSP, real‐time quantitative methylation‐specific PCR

**Figure 3:**
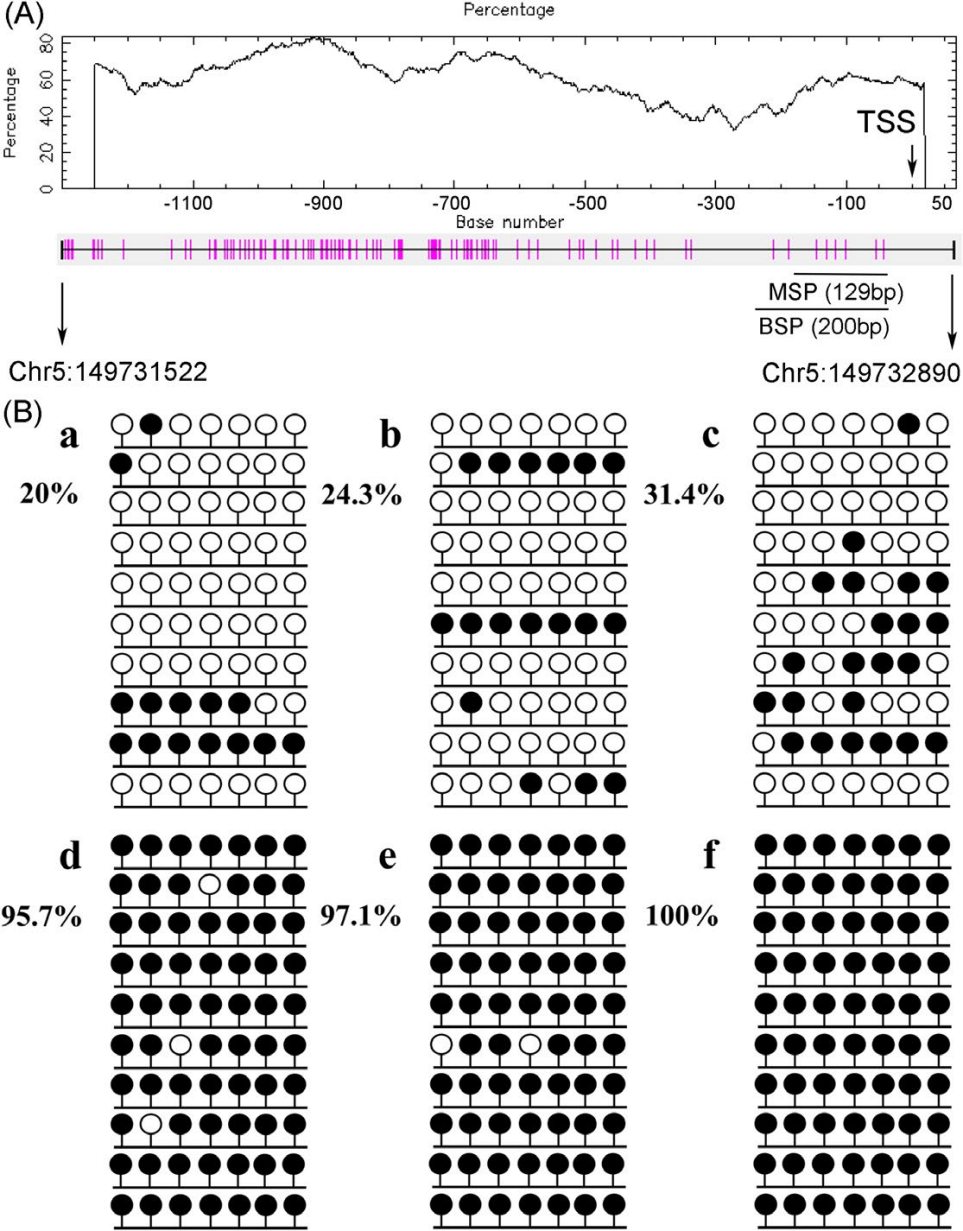
The CpG site of *MIR‐378* 5’‐flanking region and methylation density of *MIR‐378* 5’‐flanking region identified in three hypomethylated and three hypermethylated MDS patients by bisulfite sequencing. (A) diagrammatic sketches of the CpG site of *MIR‐378 5*’‐flanking region. The vertical lines represent the cytosine resides of CpGs. The black and gray arrows indicate the locations of the primers for BSP and RQ‐MSP respectively. (B) methylation density. Black lollipop: methylated CpG dinucleotide; Blank lollipop: unmethylated CpG dinucleotide. (a–c) Three hypomethylated samples; (d–f) three hypermethylated samples. The results of RQ‐MSP and bisulfite‐sequencing were significantly negative correlated (*R* *=* −.886*, p* = .019). BSP, bisulfite‐sequencing PCR; MDS, myelodysplastic syndrome; RQ‐MSP, real‐time quantitative methylation‐specific PCR

The human *MIR‐378* gene locates in chromosome 5q32, according to bioinformatics analysis, there are three CpG islands within −2.0 kb consisted in *MIR‐378* 5’‐flanking region (Xiao‐Wen et al., 2015). As was disclosed by RQ‐MSP, the unmethylated level of *MIR‐378* 5’‐flanking region was substantially higher in MDS patients (median 0.021, range 0–23.990) than that in controls (median 0.005, range 0–1.000) (*p* = .034, Figure 1). The products of RQ‐MSP for *MIR‐378* methylation and *MIR‐378* unmethylation were analyzed by agarose gel electrophoresis and the representative electrophoretogram is displayed in Figure 2. Bisulfite sequencing was further performed to examine three patients with *MIR‐378* hypomethylation and three patients with *MIR‐378* hypermethylation on the basis of RQ‐MSP, and the results are shown in Figure 3. There was a significantly negative correlation between the results of RQ‐MSP and BSP (*R* = −.886, *p* = .019). Thus, our BSP analysis confirmed the results of RQ‐MSP.

### 3.2 Association between *MIR‐378* 5’‐flanking region methylation and clinical characteristics

For clinical relevance analysis, the MDS patients were divided into two groups, the one with hypomethylated *MIR‐378* and the other without hypomethylated *MIR‐378,* based on the value of the mean plus 1 *SD* (0.252) in normal controls. Accordingly, hypomethylated *MIR‐378* was identified in 20 of 95 (21.1%) cases, however, there is no statistical significance as compared to healthy donors (6.3% [1/16], *p* = .188). No significant difference was observed in age, white blood cell counts, platelets, hemoglobin, as well as karyotypic groups between the two groups (*p* > .05). The incidence of hypomethylation of *MIR‐378* in male (15/55, 27.3%) was significantly higher than that in female (4/40, 10.0%) (*p* = .040). There is no significant difference in distribution of *MIR‐378* hypomethylaton in the FAB classifications, WHO classifications, or IPSS subgroups. It was also failed to find distinction of the five gene mutations between the two groups (*p* > .05) (Table 1).

### 3.3 Correlation between *MIR‐378* 5’‐flanking region methylation and prognosis

**Figure 4:**
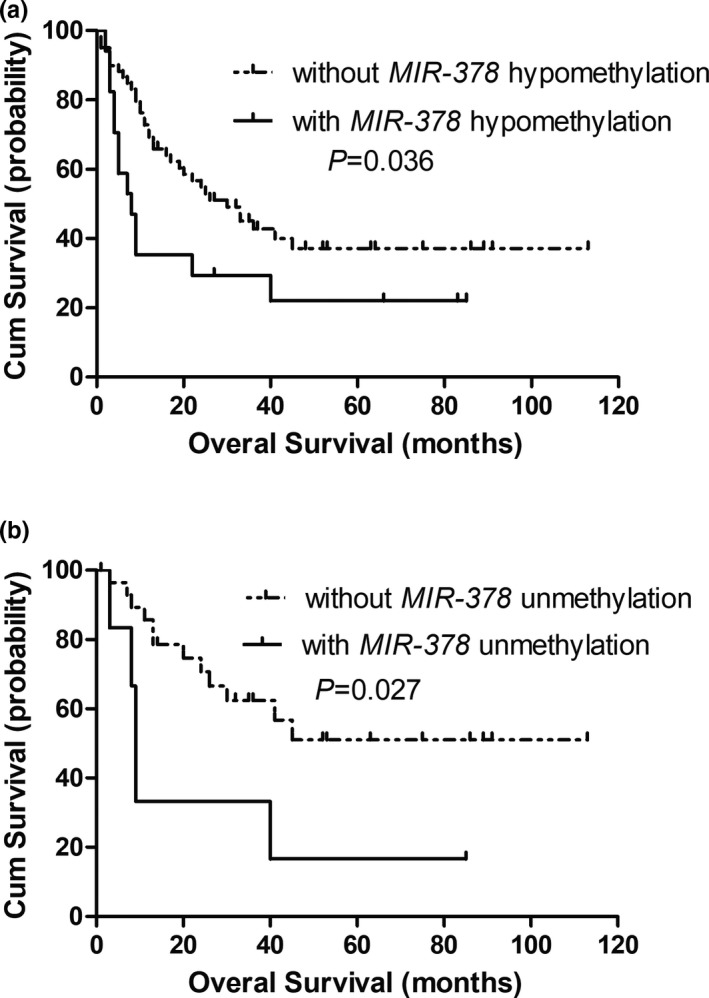
Overall survival of MDS patients. (a) All patients; (b) MDS patients <60 years. MDS, myelodysplastic syndrome

A total of 77 cases with available follow‐up data were included in our survival analysis. The median survival of all patients was about 24 months. Hypomethylated patients presented significantly worse survival compared to hypermethylated patients (median 8 vs. 30 months, respectively, *p* = .036, Figure 4a). Subgroup analysis revealed that patients with *MIR‐378* hypomethylation presented slightly worse OS (median 7 months) than those without *MIR‐378* hypomethylation (median not obtained) in cytogenetically normal patients (*p* = .152). Multivariate analysis including sex (male vs. female), IPSS (Low/Int‐1/Int‐2/High), *MIR‐378* hypomethylation (yes vs. no), five gene mutations (mt vs. wt) failed to demonstrat independent prognostic factor of *MIR‐378* hypomethylation in whole MDS patients (Table 3). However, among the age <60 years, patients with *MIR‐378* hypomethylation had significantly shorter OS (median 9 months) than those without *MIR‐378* hypomethylation (median not obtained) (*p = *.027, Figure 4b). The negative prognostic impact was also confirmed by multivariate analysis (Table 4).

**Table 3:** Multivariate analyses of prognostic factors for overall survival in MDS patients

	OS multivariate analysis		
	HR^a^	95% CI	*p* value
Sex	0.385	0.189–0.786	.009
IPSS stratification	1.525	1.076–2.161	.018
*MIR‐378* hypomethylation	1.709	0.830–3.519	.146
*IDH1/2* mutation	7.827	0.685–89.402	.098
*DNMT3A* mutation	2.530	0.758–8.443	.131
*U2AF1* mutation	1.410	0.364–5.456	.619
*SF3B1* mutation	3.240	0.898–11.693	.073

Abbreviations: CI, confidential interval; HR, hazard ratio; IPSS, International Prognostic Scoring System; MDS, myelodysplastic syndrome; OS, overall survival.

a HR >1 indicates an increased risk of an event for the first category listed.

**Table 4:** Multivariate analyses of prognostic factors for overall survival in young (age <60 years) MDS patients

	OS multivariate analysis		
	HR^a^	95% CI	*p* value
Sex	1.664	0.398–6.954	.485
IPSS stratification	4.181	1.908–9.159	.000
*MIR‐378* hypomethylation	6.619	1.694–25.864	.007
*IDH1/2* mutation	7.827	0.685–89.402	.098
*DNMT3A* mutation	17.391	1.551–194.943	.021
*U2AF1* mutation	1.410	0.364–5.456	.619
*SF3B1* mutation	31.725	2.604–386.451	.007

Abbreviations: CI, confidential interval; HR, hazard ratio; IPSS, International Prognostic Scoring System; MDS, myelodysplastic syndrome; OS, overall survival.

a HR >1 indicates an increased risk of an event for the first category listed.

## 4 DISCUSSION

Increasing numbers of microRNAs have been evidenced to participate in tumor initiation, evolution, drug‐resistance, and disease recurrence (Eitan et al., 2009; Esquela‐Kerscher & Slack, 2006; Fattore, Sacconi, Mancini, & Ciliberto, 2017; Ipn, Ng, Baharuddin, & Zakaria, 2017; Nicoloso, Spizzo, Shimizu, Rossi, & Calin, 2009; Ventura & Jacks, 2009), moreover, several of them have been confirmed to be useful tumor diagnostic and prognostic biomarkers. It is reported that *MIR‐378* had the abilities to enhance some types of cancer cell proliferation, reduce the cell apoptosis in vitro, as well as promote neoplasm growth, angiogenesis, and metastasis in vivo (Chen et al., 2012; Lee, Deng, Wang, & Yang, 2007; Ma et al., 2014; Skrzypek et al., 2013; Yu et al., 2014). Clinically, a meta‐analysis presented recently has demonstrated the prospective diagnostic value of *MIR‐378* in human cancers (Li, Shen, Li, Chen, & Chen, 2016). In AML, we firstly reported aberrant overexpression of *MIR‐378* presented in FAB‐M2 subtype, particularly in cytogenetical classification of t(8;21) chromosomal translocation (Qian et al., 2013). Our RQ‐MSP analysis correspondently shown high incidence of hypomethylation of *MIR‐378* presented in FAB‐M2 subtype, as well as for the cohort with t(8;21) chromosomal aberration (Xiao‐Wen et al., 2015). In vitro experiment, THP‐1 cells, an AML cell line, treated with a HMA 5‐aza‐dC, exhibited up‐expression of *MIR‐378* in a dose‐dependent manner (Xiao‐Wen et al., 2015), providing the evidence of methylation regulation of *MIR‐378* in AML cell line (Xiao‐Wen et al., 2015). Meanwhile, aberrant expression of *MIR‐378* has even been reported in MDS by other studies, and the distinct expression depends on the different context of cytogenetics or different stage of MDS (Erdogan et al., 2011; Merkerova et al., 2011). Here in, we highlighted *MIR‐378* methylation status in MDS, and the result demonstrated the generally hypomethylated *MIR‐378* in MDS, those were compared to normal controls. Hypomethylation of *MIR‐378* could be detected in 21% cases in our experiment. However, the distribution hypomethylation was not distinguishing either in cytogenetic classifications or in IPSS‐risk subgroups. Interestingly, our data indicated that *MIR‐378* hypomethylation occurred more frequently in male patients. This phenomenon may be attributed to the prevalence of smoking in male people in China. It is crucial that the change of DNA methylation is another contribution by tobacco smoke in cancer (Alghanim, Wu, & Mccord, 2018; Tsaprouni et al., 2014). Whether *MIR‐378* hypomethylation is correlated with smoking needs epidemiological investigation. In the present study a panel of gene mutations (*IDH1*, *IDH2*, *DNMT3A*, *SF3B1*,and *U2AF1*), genetically reported to be the important regulator in DNA methylation and RNA spliceosome and highly associated with MDS by recent studies (Adès et al., 2014; Gill, Leung, & Kwong, 2016; Lee et al., 2015; Lin et al., 2011, 2012), was analyzed by HRMA to further explore the correlation of hypomethylated *MIR‐378* and genetic changes in MDS. However, no significant difference was found in the frequencies of five gene mutations (*IDH1, IDH2, DNMT3A, SF3B1,* and *U2AF1*) between patients with and without *MIR‐378* hypomethylation.

Up‐expression rather than hypomethylation of *MIR‐378* has been disclosed to be an adverse prognosticator in AML by our former studies (Qian et al., 2013; Xiao‐Wen et al., 2015). Notably, in this study we demonstrated that hypomethylation of *MIR‐378* predicted poor outcome in MDS, particularly in patients <60 years. In our multivariate analysis, IPSS stratification, *DNMT3A* and *SF3B1* mutations could significantly predict the outcome of younger patients (<60 years), in addition to these, hypomethylation of *MIR‐378* also acts as a risk factor independently, providing helpful information for the prognosis of MDS. Further studies are needed to confirm the results before it can be used routinely as a potential marker for risk stratification in the patients with MDS.

Briefly, our study revealed that hypomethylaiton of *MIR‐378* was a common epigenetic phenomenon in MDS, and predicted poor outcome of more young patients (<60 years).

## CONFLICT OF INTEREST

None declared.

## AUTHORS CONTRIBUTION

Jiang Lin and Jun Qian designed the study; Xiang‐mei Wen, Ying‐ying Zhang and Ji‐chun Ma performed experiments; De‐hong Wu, Xiao‐wen analyzed the data, created figures, and assisted in manuscript writing; Dong‐ming Yao and Jing‐dong Zhou analyzed data; Hong Guo, Peng‐fei Wu, Xing‐li Zhang, and Hong‐chun Qiu contributed patients data.
